# Editorial: Advancements in sequencing technologies for epigenomic and transcriptomic analysis: from bulk to single-cell resolution

**DOI:** 10.3389/fgene.2026.1807544

**Published:** 2026-02-18

**Authors:** Laura Veschetti, Massimiliano Cocca, Dougba Noel Dago, Giovanni Malerba

**Affiliations:** 1 Infections and Cystic Fibrosis Unit, Division of Immunology, Transplantation and Infectious Diseases, IRCCS San Raffaele Scientific Institute, Milano, Italy; 2 Vita-Salute San Raffaele University, Milano, Italy; 3 UMR PaThLiv Inserm 1350 Université Lyon 1 (UCBL1), Lyon, France; 4 The Lyon Hepatology Institute EVEREST, Lyon, France; 5 Péléforo-Gbon-Coulibaly University, Korhogo, Côte d’Ivoire; 6 GM Lab, Department of Surgical Sciences, Dentistry, Gynaecology and Paediatrics, University of Verona, Verona, Italy

**Keywords:** epigenomics, sequencing data analysis, sequencing technologies, single-cell sequencing, transcriptomics

Over the past decade, NGS technology has transformed the way to study and explore genomics, epigenomics, and transcriptomics. Improvements in throughput, and accuracy now allow researchers to move from bulk analyses to single-cell and high-resolution approaches, uncovering cellular heterogeneity and dynamic gene regulation that were previously unattainable. These advances, however, have amplified challenges associated with the broad range of applications feeding into sequencing technologies, including sample preparation, data management, integrative analysis, and interpretation. The articles in this Research Topic collectively address these challenges, offering solutions that advance both fundamental research and translational applications.

Reliable sample preparation remains the foundation of any sequencing study. Bentz et al. present optimized high-throughput PacBio workflows for genomic DNA and Threose Nucleic Acid (TNA), demonstrating improvements in throughput, cost-efficiency, and analysis resolution. These methodological innovations illustrate how early-stage experimental improvements directly influence what can be measured and understood downstream.

As data volume and complexity grow, effective management and accessibility become increasingly critical for the researchers. Large publicly available repositories, such as The Cancer Genome Atlas (TCGA), have become essential for integrative multi-omic studies, yet accurate data extraction and preprocessing remain critical to unlock their full potential. Baumann et al. address this challenge by introducing a tool that simplifies access to TCGA datasets, thus improving data retrieval and harmonization, and enabling reproducible analyses across genomics, epigenomics, and transcriptomics studies. The work highlights the importance of precise data handling to gain robust insights from multi-omic repositories.

Computational strategies are central to interpreting high-dimensional datasets, particularly in single-cell studies where technical noise, sparsity, and batch effects complicate analysis. In this context, Elshiekh et al. review how deep learning and advanced computational models help in extracting meaningful biological signals from high-dimensional single-cell datasets, underlining the importance of integrating experimental and computational expertise to interpret complex omics data effectively. Complementing these strategies, Zhang et al. focus on transcriptional bursting models, demonstrating how modeling assumptions influence our understanding of gene expression variability and its relationship to phenotypic diversity and disease. Quantitative frameworks like these allow researchers to move from descriptive observations to mechanistic insights.

Translating sequencing insights into clinical contexts remains a translational gap. Lin et al. investigate RNA N6-methyladenosine methylation and gene expression in colorectal cancer, using an integrative transcriptome-wide approach to uncover regulatory mechanisms directly relevant to precision oncology. Together with genomic and epigenomic analyses, RNA-focused studies provide a more comprehensive view of molecular regulation and help bridge molecular insight with the clinical application of high-resolution sequencing approaches. Similarly, single-cell technologies are also approaching clinical maturity, enabling detailed characterization of patient-specific cellular heterogeneity. The translation into clinical practice requires robust, adaptable pipelines that integrate high-resolution data with computational models and curated databases, providing a pathway toward biomarker discovery and personalized therapies.

Taken together, the contributions to this Research Topic illustrate how sequencing has become the common integrative backbone of diverse high-throughput approaches, evolving from a data-generation tool into a framework for biological discovery and translational research. By advancing experimental protocols, improving data accessibility, and developing computational frameworks, these studies represent a step toward a more integrated and clinically relevant understanding of molecular biology.

